# Analysis of the Incidence of Knee Arthroscopy, Total Knee Arthroplasty (TKA), and Readmission Rates for TKA After Previous Knee Arthroscopy in the Queensland (QLD) Population

**DOI:** 10.1186/s12891-025-08521-2

**Published:** 2025-03-25

**Authors:** Sang Hyun Samuel Kang, Annie Yunser Hwang, David Ta-chun Ho, Thirumurugan Thangarajah, Lahann Wijenayake, Simon Parfit, Andrew Mclean, James Clayton

**Affiliations:** Redland Hospital, Princess Alexandra Hospital, Queensland Children’s Hospital, University of Queensland, Queensland Health, Brisbane, QLD Australia

**Keywords:** Arthroscope, Arthroplasty

## Abstract

**Background:**

In situations of osteoarthritis (OA), the therapeutic value of knee arthroscopy is still a topic of discussion that is ongoing. This study aims to produce data about the relevance of arthroscopy and total knee arthroplasty (TKA) in knee OA by assessing the overall rates of arthroscopy and subsequent conversion to TKA over a two-year period in QLD Australia within the time frame between 2008 and 2023.

**Methods:**

A retrospective cohort analysis was undertaken at Queensland (QLD) hospitals that underwent arthroscopy and TKA between 2008 and 2023. Research datasets obtained from the Centre for Health Record Linkage (CHeReL) were analyzed using negative binomial regression. An investigation was performed to determine the rates of arthroscopy admissions by year, age group, sex, and hospital system (public versus private). The TKA readmission rates were calculated during a 24-month period.

**Results:**

The results demonstrate an overall decrease in the frequency of arthroscopies from 2008 to 2022, but total knee arthroplasties (TKA) have increased by 2.79% (95% CI: 2.19 to 3.38). There was a 3.34% reduction in arthroscopy rates at private hospitals and a 0.98% drop at public hospitals (95% CI: -0.26 to 2.23). The largest significant rise in TKA rates was seen in private hospitals. The TKA procedure was performed on 14.48% of patients aged 65 and older within 24 months of knee arthroscopy. After taking sex and age into account, there was a 4.97% reduction in the occurrence of total knee TKA within 24 months following knee arthroscopy (95% CI: -5.55 to -4.40) across the board.

**Conclusions:**

While the rates of TKA are on the rise, arthroscopies and their conversions to TKA are on the decrease. Possible explanations for the persistent drop in TKA conversion rates include better patient selection, more effective non-operative care, or longer wait times for the operation.

## Introduction

Arthroscopes and arthroplasty are critical instruments and procedures in modern orthopaedic practice, enabling minimally invasive interventions and efficient joint restoration. Their implementation can considerably improve patient outcomes and recuperation durations. Surgeons have used arthroscopic operations to slow the course of OA, providing brief discomfort alleviation and extending natural knee function before pursuing more therapies [1, 2].

The current arthroscopic recommendations lack worldwide uniformity. Typically, patients with definite radiographic symptoms of OA are advised against arthroscopic debridement since there is little evidence to suggest significant symptom alleviation. The Australian Orthopaedic Association (AOA) issued a position statement in 2012, supported by significant evidence in the medical literature, indicating that arthroscopic procedures do not effectively manage symptomatic OA, regardless of the presence of radiologic signs of meniscal tears [3, 4]. In addition to randomized controlled studies, the incidence of TKA within 24 months following arthroscopy is used to assess the therapeutic effectiveness of the procedure [3, 5].

A research study conducted in 2008 looked at the age-standardized use rate of arthroscopy in the Australian population, broken down by hospital status. It also measured the proportion of patients who underwent subsequent TKA within a 24-month period [6]. The definition of TKA in this study included every aspects of knee arthroplasty as a grouped terminology taking account of unicompartmental knee arthroplasty (UKA) and patellofemoral knee arthroplasty (PF). This study found that the rate of knee arthroscopies has remained stable, but the percentage of patients requiring a TKA within 24 months of a knee arthroscopy has decreased in the age group most prone to degenerative changes in the knee [6]. This study looked at demographic groupings in New South Wales (NSW), emphasizing that results may vary by state due to differences in geography and socioeconomic variables. Furthermore, the publication of further randomized control studies and modifications to the AOA statement may have altered trends in these rates [1, 3, 7].

Following its publication, more randomized controlled studies occurred, as did the AOA statement, which may have affected knee arthroscopy and TKA rates [7]. This study is a continuation of the 2008 research undertaken in New South Wales, with an emphasis on trend evaluation using more current data from QLD populations from 2008 to 2023. The aim of this study is to look at the rates of knee arthroscopies and the fraction of these patients that require follow-up TKA within 24 months.

## Methods

Harris et al. [6] provide detailed documentation of the process. The method makes use of comparable data sources from QLD throughout several time periods, with a focus on surgery and conversion rates. Minor changes were made to the methods outlined by Harris et al. [6]. The data used to compile these revisions comes from the QLD Hospital Admitted Patient Data Collection (QHAPDC) and the Statistical Services Branch (SSB) of QLD Health, specifically on arthroscopy and TKA that took place in the state between 2008 and 2023. QHAPDC collects detailed data on admitted patient episodes of care from all QLD hospitals, public and private, as well as day-procedure facilities. According to the Australian Classification of Health Intervention procedure codes, Table 1 displays the operations performed during TKA and knee arthroscopy episodes. The TKA as mentioned above TKA is the umbrella terminology taking account of unicompartment and patellofemoral joint. Clinical indications for TKA is for end stage management of knee conditions after knee arthroscope except for those relating to the ligament restoration. All arthroscopy codes were used, except for those relating to ligament restoration. It indicates that a definite diagnosis is necessary for the indication of arthroscopy. Given the lack of mechanical symptoms, it can be inferred that the surgery was performed to address discomfort related to the final diagnosis of osteoarthritis. Consequently, although the reporting lacks precision, it is not anticipated that this will undermine the validity of the conclusions. The Queensland Health Master Linkage File (QLDHMLF) allowed for the systematic identification of care episodes for the same patients across many institutions in QLD. According to the QLD Health info-bank demographics, age-specific population estimates were derived as of 31 December for each year. This was done since these dates mark the midpoint of each financial year of hospitalization.

**Table 1:** Procedure codes for data extraction

Total knee arthroplasty (TKA)
49,517–00 Hemiarthroplasty of knee
49,518–00 Total arthroplasty of knee, unilateral
49,519–00 Total arthroplasty of knee, bilateral
49,521–00 Total arthroplasty of knee with bone graft to femur, unilateral
49,521–01 Total arthroplasty of knee with bone graft to femur, bilateral
49,521–02 Total arthroplasty of knee with bone graft to tibia, unilateral
49,521–03 Total arthroplasty of knee with bone graft to tibia, bilateral
49,524–00 Total arthroplasty of knee with bone graft to femur and tibia, unilateral
49,524–01 Total arthroplasty of knee with bone graft to femur and tibia, bilateral
49,534–01 Total Replacement arthroplasty of patellofemoral joint of knee
**Arthroscopy**
Arthroscopy excluding meniscectomy
49,557–00 Arthroscopy of knee
49,557–01 Arthroscopic biopsy of knee
49,558–00 Arthroscopic debridement of knee
49,558–01 Arthroscopic chondroplasty of knee
49,558–02 Arthroscopic osteoplasty of knee
49,559–00 Arthroscopic chondroplasty of knee with multiple drilling or implant
49,560–00 Arthroscopic removal of loose body of knee
49,560–01 Arthroscopic trimming of ligament of knee
49,560–02 Arthroscopic lateral release of knee
49,561–00 Arthroscopic lateral release of knee with debridement, osteoplasty or chondroplasty
49,561–02 Arthroscopic removal of loose body of knee with debridement, osteoplasty or chondroplasty
49,562–00 Arthroscopic lateral release of knee with chondroplasty and multiple drilling or implant
49,562–02 Arthroscopic removal of loose body of knee with chondroplasty and multiple drilling or implant
49,563–00 Arthroscopic repair of meniscus of knee
49,566–00 Arthroscopic synovectomy of knee
49,503–06 Arthroscopic chondroplasty of knee with multiple drilling or implant
Meniscectomy
49,557–02 Arthroscopic excision of meniscal margin or plica of knee
49,560–03 Arthroscopic meniscectomy of knee
49,561–01 Arthroscopic meniscectomy of knee with debridement, osteoplasty or chondroplasty
49,562–01 Arthroscopic meniscectomy of knee with chondroplasty and multiple drilling or implant

Data custodian and QLD Population and Health Services Research Ethics Committee (HREC) clearances were received, and the SSB then submitted lists with de-identified aggregated count data. The MLF does not cover private hospitals before June 30, 2008; hence, the research focuses on the period from July 1, 2008, to December 31, 2023, incorporating the most recent available data. The number of knee arthroscopies and total knee arthroplasties (TKA) was recorded and categorized by age, biological sex, year, and hospital sector (private versus public). The information on the indications for knee arthroscopy was shown to be insufficient, reducing its credibility for reporting purposes. Quality studies of MLF linkage often show false positive linking rates of 5 per 1,000 records (0.5%) or below. The rates of TKA and knee arthroscopy were determined per 100,000 people aged 18 and above. The rate of TKA within 24 months post knee arthroscopy was estimated by dividing the total number of TKAs performed during that timeframe by the total number of knee arthroscopies performed every year. Negative binomial regression analyses were used to determine the percentage change in rates. The key variable under examination was the year, with age and sex being considered as secondary factors. To assess the rate of change as a percentage and to deal with data over-dispersion, negative binomial regression models were used. The logarithm of the QLD population for each year is used as an adjustment to account for population fluctuations throughout time.

## Results

### Rates of knee arthroscopy by year and hospital status

**Fig. 1:**
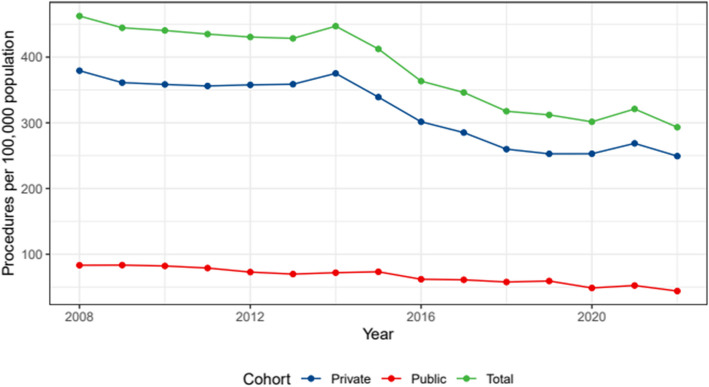
Total, public and private knee arthroscopy rates per 100,000 population between 2008 and 2022

Table 2 includes descriptive information for patients who have had knee arthroscopy and TKA. Between 2008 and 2022, the rate of knee arthroscopy decreased from 462.5 to 293.2 per 100,000 individuals. This is a change of −3.54% (95% CI: −4.20 to 2.89). (Fig. 1). Public rates fell from 83.2 per 100,000 people in 2008 to 43.9 per 100,000 in 2022, representing a rate drop of −4.32% (95% confidence interval: −5.02 to −3.61). In comparison, private hospital rates declined from 379.3 per 100,000 people in 2008 to 268.7 per 100,000 in 2022, suggesting a rate change of −3.38% (95% CI: −4.06 to −2.70). Between 2008 and 2022, the ratio of private to public knee arthroscopies in QLD increased by 5.8:1, up from 4.76:1.

**Table 2:**
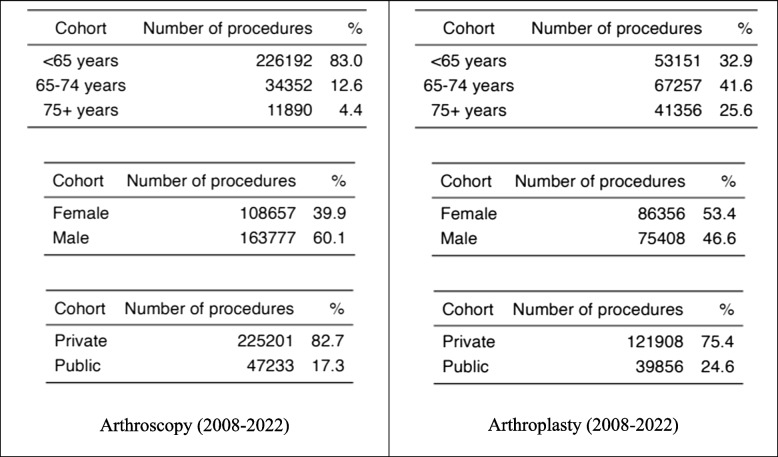
Descriptive Statistics for all arthroscopy and arthroplasty patients, QLD 2008 –2022

### Age-specific rates of knee arthroscopy

**Fig. 2:**
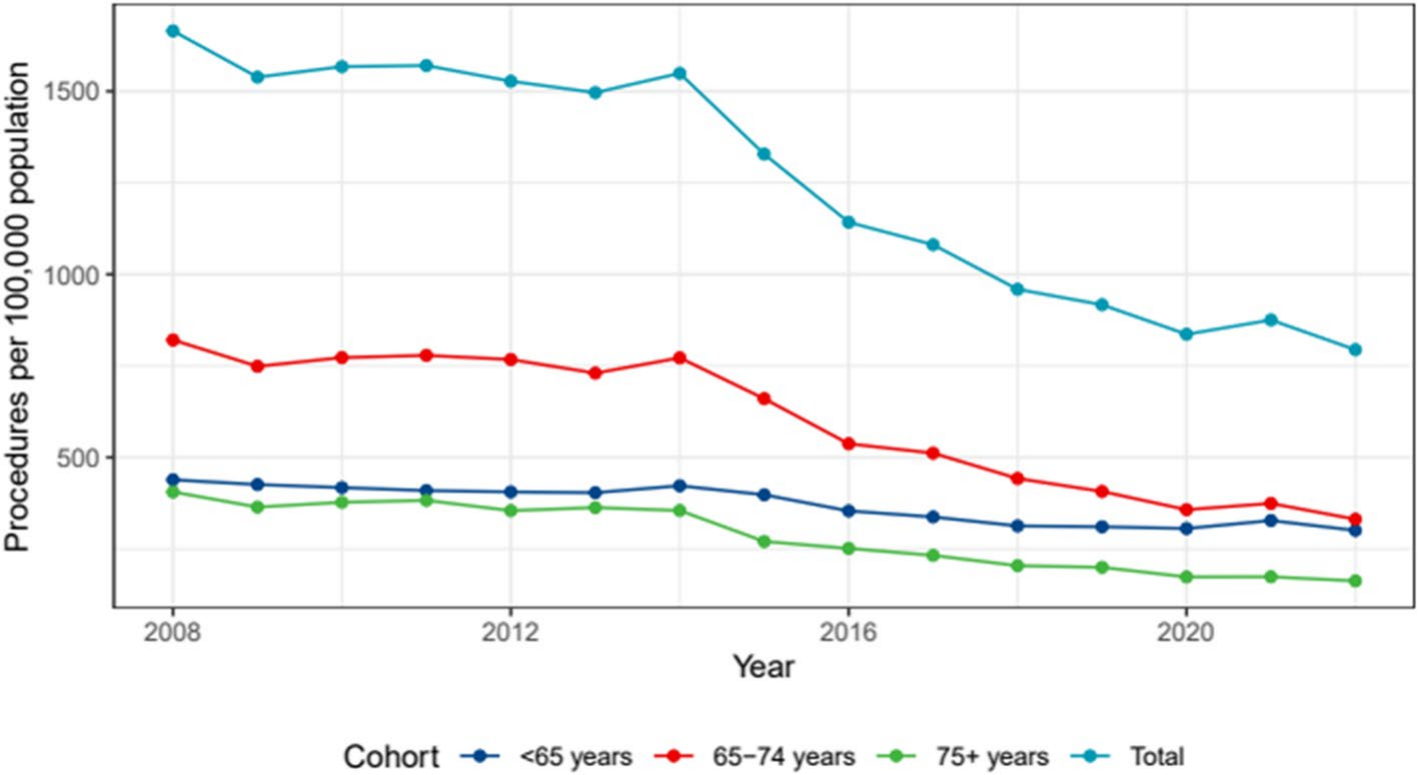
Knee arthroscopy rates per 100,000 population between 2008 and 2022 by age group

The research period includes an investigation of the rate of knee arthroscopy by age (Fig. 2). After adjusting for sex, there was a significant shift in the frequencies of knee arthroscopy among the 65–74 age group across the observed time period (Table 3).

**Table 3:** Changes in Knee Arthroscopy rate between 2008 and 2022 for each age group

Cohort	Change	95% CI Lower Bound	95% CI Upper Bound
< 65 years	−2.92	−3.85	−2.00
65–74 years	−7.07	−8.03	−6.12
75 + years	−7.24	−8.25	−6.23

### Hospital status and yearly rates of knee arthroplasty

**Fig. 3:**
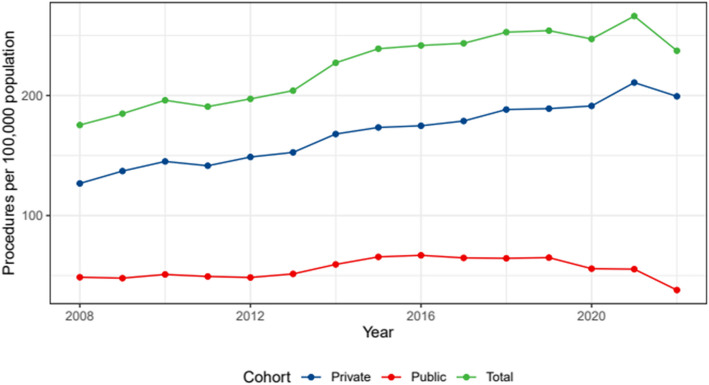
Total rates, public and private TKA per 100,000 population between 2008 and 2022

The rate of TKA increased by 2.79% (95% CI: 2.19 to 3.38%) between 2008 and 2022, from 175.37 to 237.2 per 100,000 individuals. (Fig. 3). In 2008, the rate in public hospitals was 48.6 per 100,000 people, and it has stayed reasonably consistent at 37.9 per 100,000 people in 2022. The rate of change was 0.98% (95% CI: −0.26 to 2.23). In private hospitals, the rate increased significantly, rising from 126.7 per 100,000 people in 2008 to 210.7 per 100,000 people in 2022, representing a 3.34% rise (95% CI: 2.11 to 4.58). In 2022, the ratio of private TKA to public TKA in QLD increased to 5.26:1, up from 2.6:1 in 2008.

### Age-related rates of TKA

**Fig. 4:**
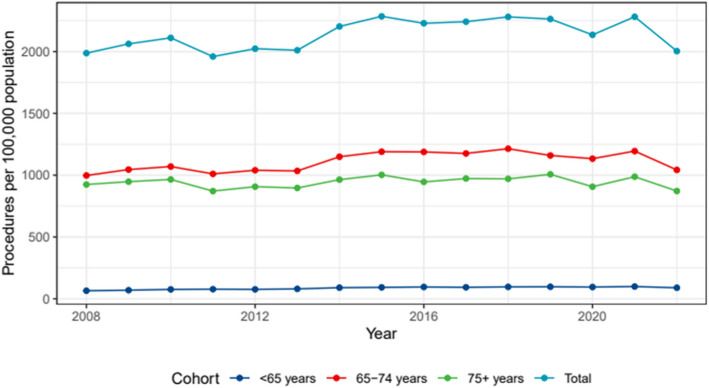
TKA rates by age group per 100,000 individuals between 2008 and 2022

The study also looked at TKA rates by age group (Fig. 4). After correcting for sex, there were significant changes in TKA rates across time among different age groups (Table 4).

**Table 4:** Changes in Knee Arthroplasty rate between 2008 and 2022 for each age group

Cohort	Change	95% CI Lower Bound	95% CI Upper Bound
< 65 years	2.62	2.00	3.25
65–74 years	0.97	0.35	1.59
75 + years	0.16	−0.47	0.80

### Readmission rates following knee arthroscopy for the initial TKA within 24 months

From 2008 to 2021, the incidence of initial TKA performed within twenty-four months after knee arthroscopy is shown in Table 5. When age and sex were included, the overall readmission rate for TKA decreased significantly. There was a −3.93% decline (95% CI: −4.76 to −3.06) and a −4.19% decline (95% CI: −4.91 to −3.47) among patients aged 65 and above in private hospitals. For public patients, the change in rate was −9.26% (95% CI: −10.82 to 7.70), and the conversion rates were stable. The 24-month follow-up data was not available beyond 2021; hence the conversion rate to TKA could not be estimated after that year.

**Table 5:** Rate of readmission for Primary TKA within 24 months of knee arthroscopy

		**2008**	**2009**	**2010**	**2011**	**2012**	**2013**	**2014**	**2015**	**2016**	**2017**	**2018**	**2019**	**2020**	**2021**	**Total**
**All Ages**	**TKA within 2 years of knee arthroscopy**	937	922	828	754	754	715	722	564	458	434	396	335	369	333	8,521
	**Knee arthroscopy**	14,809	14,373	13,999	13,651	13,488	13,468	14,077	12,774	11,193	10,648	9,757	9,553	9,226	9,801	170,817
	**Proportion (%)**	**6.3**	**6.4**	**5.9**	**5.5**	**5.6**	**5.3**	**5.1**	**4.4**	**4.1**	**4.1**	**4.1**	**3.5**	**4.0**	**3.4**	**5.0**
**All Ages (Private)**	**TKA within 2 years of knee arthroscopy**	782	801	690	629	634	610	618	467	394	375	348	296	338	308	7,290
	**Knee arthroscopy**	12,210	11,718	11,383	11,080	11,162	11,202	11,699	10,246	8,998	8,487	7,719	7,512	7,538	7,952	138,906
	**Proportion (%)**	**6.4**	**6.8**	**6.1**	**5.7**	**5.7**	**5.4**	**5.3**	**4.6**	**4.4**	**4.4**	**4.5**	**3.9**	**4.5**	**3.9**	**5.2**
**All Ages (Public)**	**TKA within 2 years of knee arthroscopy**	155	121	138	125	120	105	104	97	64	59	48	39	31	25	1,231
	**Knee arthroscopy**	2,599	2,655	2,616	2,571	2,326	2,266	2,378	2,528	2,195	2,161	2,038	2,041	1,688	1,849	31,911
	**Proportion (%)**	**6.0**	**4.6**	**5.3**	**4.9**	**5.2**	**4.6**	**4.4**	**3.8**	**2.9**	**2.7**	**2.4**	**1.9**	**1.8**	**1.4**	**3.9**
**Age 65 + **	**TKA within 2 years of knee arthroscopy**	462	423	400	364	401	362	393	271	239	194	202	178	172	142	4,203
	**Knee arthroscopy**	2,460	2,313	2,379	2,462	2,447	2,494	2,595	2,120	1,833	1,709	1,535	1,445	1,318	1,352	28,462
	**Proportion (%)**	**18.8**	**18.3**	**16.8**	**14.8**	**16.4**	**14.5**	**15.1**	**12.8**	**13.0**	**11.4**	**13.2**	**12.3**	**13.1**	**10.5**	**14.8**

## Discussion

Following Harris et al.'s study, other articles have arisen on the general trends in knee arthroscopy and TKA in Australia. In 2012, a study done in Victoria found that the number of elective knee arthroscopies performed in hospitals around the region had decreased [2]. A 2019 study letter evaluated process data from the Australian Institute of Health and Welfare, citing the findings of Harris et al. [6, 8]. The study letter gives more evidence for the drop in knee arthroscopy and emphasizes the lengthy timescale necessary for the AOA policy statement to be resolved [2, 3, 8]. The study's main strength is its methodological agreement with the Harries et al. research, as well as its specific applicability to the QLD population using state-wide data across a 15-year period.

In 2022, the incidence of knee arthroscopy in QLD was found to be lower than the statistics reported by Harris et al. in 2008 for New South Wales [6]. The data shows a general trend of declining arthroscopy rates from 2002 to 2015 in Florida, USA [9]. The yearly absolute number of knee arthroscopies performed in Sweden decreased significantly from 2002 to 2016 [10].

Researchers in New South Wales found no statistically significant shift in the prevalence of knee arthroscopy between the years 2000 and 2008 [6]. This is in stark contrast to QLD in 2022, where rates have fallen sharply, particularly after 2014. This might suggest the significance of the recent AOA statement on the correct use of knee arthroscopy in instances of OA. Nonetheless, prior to the publishing of the statement, there were studies demonstrating the ineffectiveness of arthroscopy as a general therapy for OA [8, 9, 11, 12]. The magnitude of the decline is unclear.

Examining data from several nations reveals that Sweden's rate fell significantly in 2012 [10]. During the 15-year study period in QLD, the incidence of knee arthroscopy surgeries decreased significantly in both public and private healthcare facilities. This is in contrast to the rates reported in New South Wales, where knee arthroscopy rates in public hospitals fell significantly during a 7.5-year period, but rates in private institutions stayed relatively consistent. The rate reported in private hospitals was noticeably higher. In 2022, private hospitals in QLD maintained high rates. The drop might be related to the interaction between health care services and larger economic variables. Individuals aged 65 to 75 had a greater proportionate reduction in arthroscopy procedures, although those over 75 had a lower overall number of surgeries.

The emergence of high-quality research on the efficacy of knee arthroscopy, together with the previous national AOA statement, has changed Australian healthcare practice by improving the clarity of indications for knee arthroscopy, resulting in fewer needless problems [2, 4, 8]. Even when there is sufficient data, changing practices clearly takes time.

Building on Harris et al.'s findings, we investigated the incidence of TKA in QLD. The total rate of TKA increased significantly throughout the 15-year study period. Although there was a progressive increase, a brief period from 2019 to 2020 suggested a probable fall in rates, a pattern that was also observed on a worldwide scale. We hypothesize that the current reduction is linked to the Covid-19 epidemic [13]. TKA is used as a last resort for persistent OA, with persons under 65 having the lowest occurrence of the surgery, with no significant variations in the rate. Nonetheless, rates increased significantly among people over the age of 65. This is consistent with previous observations of a naturally increased frequency of OA among the elderly [5, 13]. Some elderly patients may not benefit from arthroscopy due to the high rate of TKA conversion within 24 months in the 65 and older age group [11]. Throughout the trial, the conversion rate from arthroscopy to TKA has steadily decreased. This is in line with the consensus reached by Harris et al., who postulated that extended waiting periods for TKA, improvements in non-operative care, or better patient selection all have a role in the ongoing decline of the conversion rate to the procedure.

Given that the datasets and analytical methodologies used in this investigation are the same, several limitations are similar to those noted by Harris et al. [6]. The operation, regardless of whether it was an arthroscopy, was limited by its dependence on administrative databases, which might contain inadequate patient information and coding mistakes. Harris et al. also noted that selection bias might result from missing procedure codes. Gaining access to the data custodian's rates of missing process codes was not feasible. Other factors impacting coding decisions were medical practices, insurance laws, and patient presentations, among others [6]. It is plausible to assume that some patients may have undergone arthroscopy and TKA on the opposite side of their affected leg since the datasets did not indicate which side was affected. To overcome this constraint, we evaluated the findings of Harris et al., who conducted research on independent institutions concurrently with large studies. Out of the 42 instances that were successfully treated with knee arthroscopies and TKA, only two were contralateral [6]. Currently conducted QLD studies have likely exaggerated the rate of TKA conversion. It is probable that the reason for knee arthroscopy influences the chance of a conversion to TKA. The documentation of the causes for knee arthroscopy was poor, thus we were unable to provide that information.

Despite the study's limitations, we investigated the conversion rate, which confirmed the ineffectiveness of arthroscopy [11, 12]. TKA is the final treatment for end-stage OA of the knee. This study will look at the revision rate of TKA in combination with previous arthroscopic surgeries, which might provide a better knowledge of the efficacy of knee arthroscopes. The QHAPDC data, together with the master linkage, may be used to calculate the number of revisions for TKA following earlier TKA and arthroscopy, allowing for more study into the quality of knee arthroscopes.

## Conclusion

Over a 15-year period, the rate of knee arthroscopy in both the private and public sectors in QLD has decreased significantly, consistent with global trends. The present conversion rate is lower than that reported by Harries et al. in 2008, and it is expected to fall more over time. In light of the new data and the AOA policy statement, implementing evidence-based practice will require a time of adjustment, increased awareness, and adherence by surgeons to reach complete establishment.

## Data Availability

Availability of data and materials- The data used to support the findings of this study are available in the text and can be procured from the corresponding author upon request.

## Abbreviations

OA: Osteoarthritis
TKA: Total knee Arthroplasty
CheReL: Centre for Health Record Linkage
AOA: Australian Orthopaedic Association
QHAPDC: Queensland Hospital Admitted Patient Data Collection
SSB: Statistics Service Branch
(HREC): Health Services Research Ethics Committee
QLDHMLF: Queensland Master Linkage File
QLD: Queensland
NSW: New South Wales
